# Supplementary material for: Case Report: Long-Term Survival of a Patient with Cerebral Metastasized Ovarian Carcinoma Treated with a Personalized Peptide Vaccine and Anti-PD-1 Therapy
Source: Vaccines (Basel). 2024 Apr 9;12(4):397. doi: 10.3390/vaccines12040397 (PMC11053407; doi:10.3390/vaccines12040397)
Supplement: Supplementary file 1 [file vaccines-12-00397-s001.zip › vaccines-2940135-supplementary.pdf]

Supplementary Figures

Supplementary Figure S1:

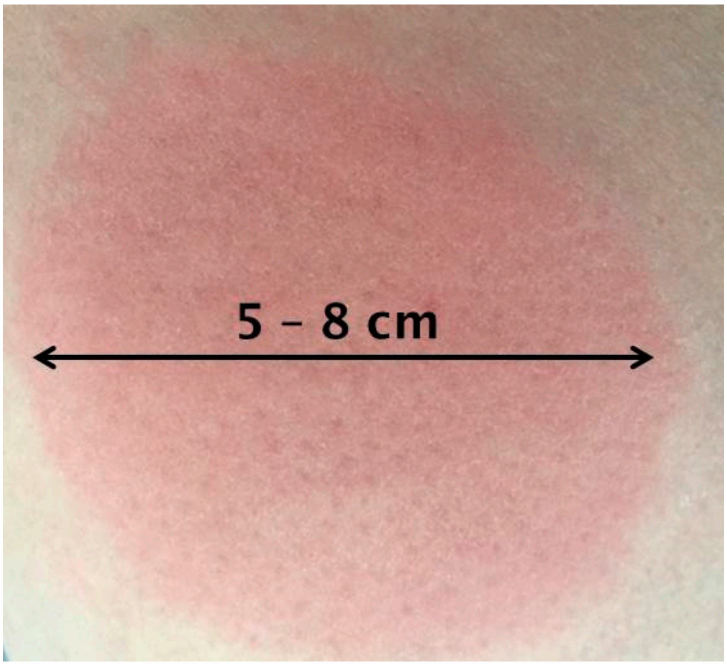

Supplementary Figure S1: Normal skin reaction several hours after injection of peptides and GM-CSF.

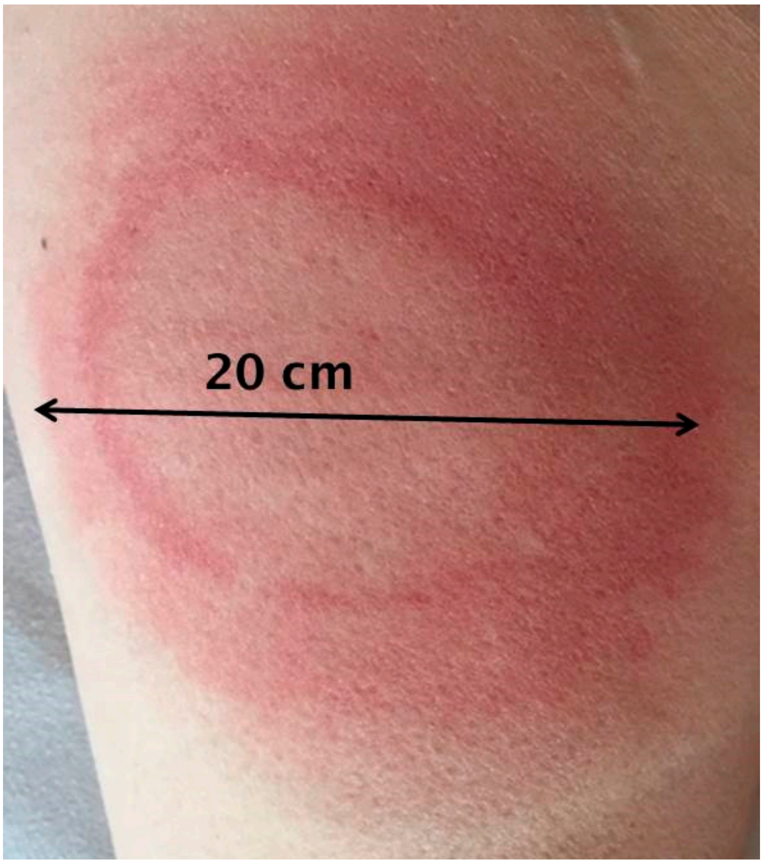

Supplementary Figure S2: Pronounced skin reaction of the patient after injection of peptides and GM-
CSF 3 hours after vaccination no. 10 (6 months after first injection).
